# Individual and Population Level Effects of Partner Notification for *Chlamydia trachomatis*

**DOI:** 10.1371/journal.pone.0051438

**Published:** 2012-12-12

**Authors:** Christian L. Althaus, Janneke C. M. Heijne, Sereina A. Herzog, Adrian Roellin, Nicola Low

**Affiliations:** 1 Institute of Social and Preventive Medicine (ISPM), University of Bern, Bern, Switzerland; 2 Department of Statistics and Applied Probability, National University of Singapore, Singapore, Singapore; Harvard School of Public Health, United States of America

## Abstract

Partner notification (PN or contact tracing) is an important aspect of treating bacterial sexually transmitted infections (STIs), such as *Chlamydia trachomatis*. It facilitates the identification of new infected cases that can be treated through individual case management. PN also acts indirectly by limiting onward transmission in the general population. However, the impact of PN, both at the level of individuals and the population, remains unclear. Since it is difficult to study the effects of PN empirically, mathematical and computational models are useful tools for investigating its potential as a public health intervention. To this end, we developed an individual-based modeling framework called Rstisim. It allows the implementation of different models of STI transmission with various levels of complexity and the reconstruction of the complete dynamic sexual partnership network over any time period. A key feature of this framework is that we can trace an individual’s partnership history in detail and investigate the outcome of different PN strategies for *C. trachomatis*. For individual case management, the results suggest that notifying three or more partners from the preceding 18 months yields substantial numbers of new cases. In contrast, the successful treatment of current partners is most important for preventing re-infection of index cases and reducing further transmission of *C. trachomatis* at the population level. The findings of this study demonstrate the difference between individual and population level outcomes of public health interventions for STIs.

## Introduction

Partner notification (PN, also known as contact tracing) is an integral part of managing several sexually transmitted infections (STIs). The process of PN for curable STIs includes informing sexual partners of infected people of their exposure, administering presumptive treatment and providing advice about the prevention of future infection [1]. PN has multiple objectives and operates at both individual and population levels [2]. One objective is the identification of new infected index cases who can be treated through individual case management. Another objective is to reduce infection prevalence by preventing onward transmission in the population. While PN is often described as an effective control intervention for different STIs, the relative effects at the level of individuals and the population are not well understood.

PN is widely used for *Chlamydia trachomatis* infections. *C. trachomatis* is the most common bacterial STI in many developed countries and is primarily found among sexually active young adults [3]. The majority of infections is asymptomatic and remains undiagnosed. Treatment and prevention of *C. trachomatis* are of particular importance to women since infection can lead to serious reproductive tract complications [4]. The importance of notifying current partners of infected index cases has been illustrated in both reviews of empirical studies [5] and modeling studies [6], [7] where PN has been shown to reduce the probability of re-infection of index cases. Notification of previous partners of index cases is also recommended, particularly as part of screening programs that aim to limit transmission and to reduce the overall prevalence of *C. trachomatis* in the population [8]. If PN is applied as an integral part of a screening intervention, it is expected to result in a more substantial reduction in the prevalence of *C. trachomatis* than would be expected by screening alone [9], [10]. However, we are not aware of any empirical studies that have reported the effect of PN on the population prevalence of *C. trachomatis*.

Several countries recommend different PN look-back periods because there is still uncertainty about the most appropriate strategy. For example, the US Centers for Disease Control and Prevention (CDC) recommends notifying partners with whom the index case has had sexual contact within the previous 60 days [11]. If no sexual contact occurred during this period, the most recent partner should be notified. Zimmermann-Rogers et al. [12] had previously pointed out that a PN period of 180 days or more would help to identify more infected cases. The UK National Guideline for the Management of Genital Tract Infection with *C. trachomatis* recommends notifying partners of an asymptomatic index case within a period of 6 months [13]. The same is standard in Sweden, but recommendations from the National Board of Health and Welfare might change, based on a recent study that found that extending PN periods could improve the identification of new *C. trachomatis* cases [14].

Mathematical and computational models for detailed examination of the effects of PN on the identification of new index cases and the reduction in onward transmission need to allow partnerships to be represented explicitly and an individual’s partnership history to be documented beyond the current partner. The impact of PN has been investigated as a general concept [15], [16] and for specific bacterial STIs such as *Neisseria gonorrhoeae* and *C. trachomatis* [9], [10], [17], [18]. These studies did not consider a dynamic sexual partnership network and/or did not follow the partnership history over a prolonged look-back period, however. Complex PN strategies that involve tracing both current and previous partners of index cases need to be investigated using stochastic, individual-based models. This structure allows partnerships to be represented explicitly, a dynamic sexual partnership network to be reconstructed over any given time period, and an individual’s partnership history beyond the current partner to be kept on record [19]. A disadvantage of existing individual-based models is that they were usually designed to address one specific research question [20]–[22] and can often not be readily adapted to different situations. In contrast, deterministic population-based models, which are based on ordinary differential equations (ODEs), offer great flexibility in altering model assumptions and for model parameterization. These models, however, cannot track individuals so their potential for studying PN interventions is limited. A modelling framework that combines the tractability of ODE models with the properties of individual-based models would be a powerful tool for examining the impact of different assumptions about model structure on the effects of PN. The importance of comparing different models and assumptions has previously been shown for chlamydia screening [8] and human papillomavirus vaccination [23].

In this paper, we present a novel, stochastic, individual-based modeling framework called Rstisim (from R STI Simulator), which allows the implementation in an individual-based manner of models of STI transmission that are described by ODEs. We apply and compare three basic models of *C. trachomatis* transmission with different assumptions about the sexual partnership dynamics. Based on these models, we investigate some general properties of PN for *C. trachomatis*. Two strategies of PN are considered: one in which partners can be notified in order of their recency, and one in which partners within a certain time period can be notified. Together, they allow us to draw some general conclusions about the effects of PN for *C. trachomatis* at both the level of individuals and the population level.

## Results

### Modeling Sexual Contacts

**Figure 1:**
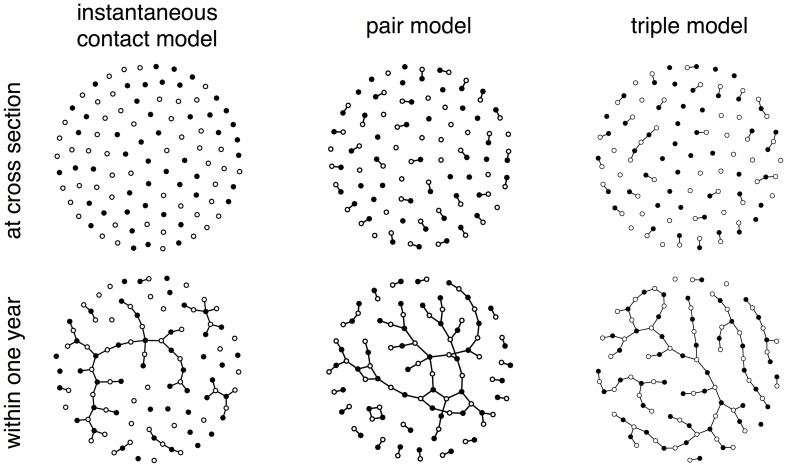
Sexual partnership networks from the three individual-based models. The ‘instantaneous contact’ model has no connectivity at cross-section but exhibits variation and connected components of different size within a period of one year. The ‘pair model’ and ‘triple model’ illustrate the connectivity at cross-section and larger connected components during a period of one year. Note that in all three models, the average number of new partnerships formed within one year is equal. For illustrative purposes, the population size was limited to 100, resulting in higher connected networks compared to larger population sizes. Different sexes are indicated by filled and empty circles.

We first derive the deterministic, population-based descriptions (ODEs) of three different models that describe heterosexual partnership dynamics with increasing levels of complexity (see *Methods*). These models are then implemented at an individual level in Rstisim, which allows the sexual partnership network over different time periods to be reconstructed. The instantaneous contact model is based on the assumption that sexual contacts happen instantaneously [24], but this model cannot account for a sexual partnership network at cross-section. In contrast, the pair model assumes that women and men form partnerships that last for a certain period [25]. Sex acts that might lead to transmission of the infection occur throughout the duration of the partnership. The triple model accounts for the fact that individuals can have two sexual partnerships at the same time (concurrency). Despite the simple nature of the models, they exhibit rich dynamics, particularly in the case of the triple model, where chains of contacts can occur at cross-section (Fig. 1). In all models, closely connected groups or bigger circular structures emerge over a period of one year.

### Number of Sex Partners and Chlamydia Transmission

The models were parameterized to reflect the sexual behavior of the general population of young adults by using data from Britain’s second National Survey of Sexual Attitudes and Lifestyles (Natsal-2) for 16–25 year old women and men [26]. All three models are adjusted so that the total number of new heterosexual contacts or partnerships and the total number of realized sex acts are equal (Table 1). For the triple model, we define the level of concurrency, 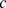, as the ratio of individuals that have two partnerships to all individuals in a partnership. By varying the level of concurrency between 0–100% we found that 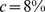 provides the best match between the simulations and data from Natsal-2 describing the gaps and overlaps between sexual partnerships (Fig. S1). Assuming 8% concurrency at cross-section in the triple model results in a cumulative incidence of concurrency of 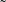
15% over the last year. This is in line with the Natsal-2 estimate for 16–24 year old women (15.2%, 95% confidence interval (CI) 12.7, 18.1%) but slightly lower than for men (20.8%, 95% CI 17.8, 24.3%) [26]. The per sex act transmission probabilities of *C. trachomatis* were calibrated to obtain an endemic prevalence of 3% and are in good agreement with empirical estimates [27]–[29]. The transmission probabilities decrease with increasing complexity of the models (Table 1) since both re-infection and concurrency facilitate the spread of STIs [6], [30].

**Table 1:** Parameters that determine the dynamics of sexual partnerships and the transmission of *C. trachomatis*.

	Instantaneous	Pair	Triple
	contact model	model	model
**Assumed parameters**			
Mean number of new heterosexual partnershipsper individual [26]	1.04 y^−1^	1.04 y^−1^	1.04 y^−1^
Mean number of total heterosexual partnershipsper individual [26]	–	1.70 y^−1^	1.70 y^−1^
Level of concurrency, *c*	–	–	8%
Proportion of individuals that are in a partnershipat cross-section	0%	67%	62%
Frequency of sex acts, *f*	–	1 per week	1 per week
Mean duration of infection, 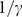 [6], [39]	1 y	1 y	1 y
Prevalence of *C. trachomatis*, 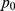 [56]	3%	3%	3%
**Derived parameters**			
Contact or pair formation rate, *ρ*	0.52 y^−1^	1.56 y^−1^	1.36 y^−1^
Relative probability of accepting a partnershipif already in a pair, *α*	–	–	0.28
Mean duration of partnership, 1/*σ*	–	0.65 y	0.65 y
Average number of sex acts per partnership, *n*	35	35	35
Transmission probability per sex act, *π*	15%	11%	9%

The different models (instantaneous contact, pair and triple model) are adjusted so that the total number of newly formed contacts or partnerships and the total number of realized sex acts are equal. To achieve the same steady-state prevalence of *C. trachomatis*, the transmission probability is varied between the models while keeping the mean duration of the infection the same. This ensures that the incidence of *C. trachomatis* infection is equal in all models. The level of concurrency at cross-section is defined as the ratio of individuals that have more than one partnership to all individuals in a partnership. Some parameters are given as rounded values.

### Partner Notification Strategies

**Figure 2:**
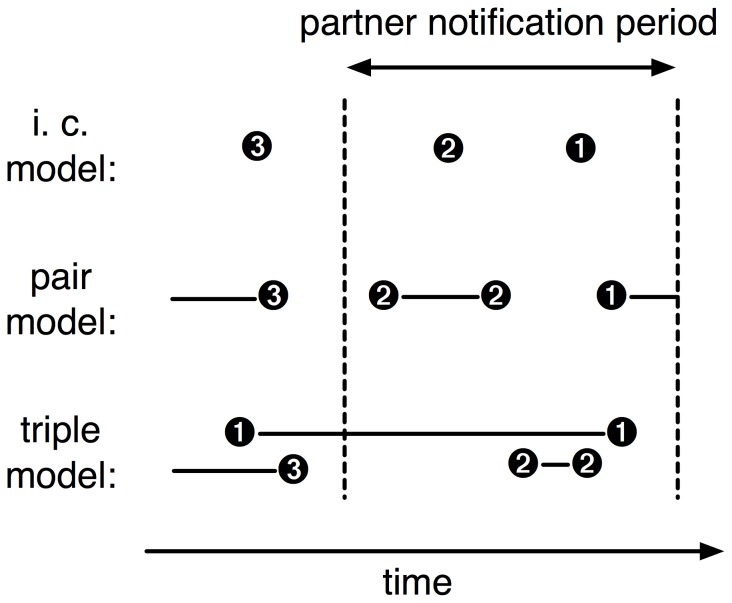
Partner notification strategies. An illustrative example of an individual’s history of contacts or partnerships is shown for each model. One strategy is to notify partners of an index case in order of their recency (time since their partnership ended). Another strategy is to notify all partners from a certain time period.

The key reason to implement the models in our individual-based modeling framework is to be able to follow an individual’s history of current and previous partners (Fig. 2). The instantaneous contact model and the pair model describe serial monogamy. The triple model illustrates complex partnership dynamics in which a new partnership can replace a previous one, or where short episodes of concurrency can occur.

#### Individual level effect of partner notification

**Figure 3:**
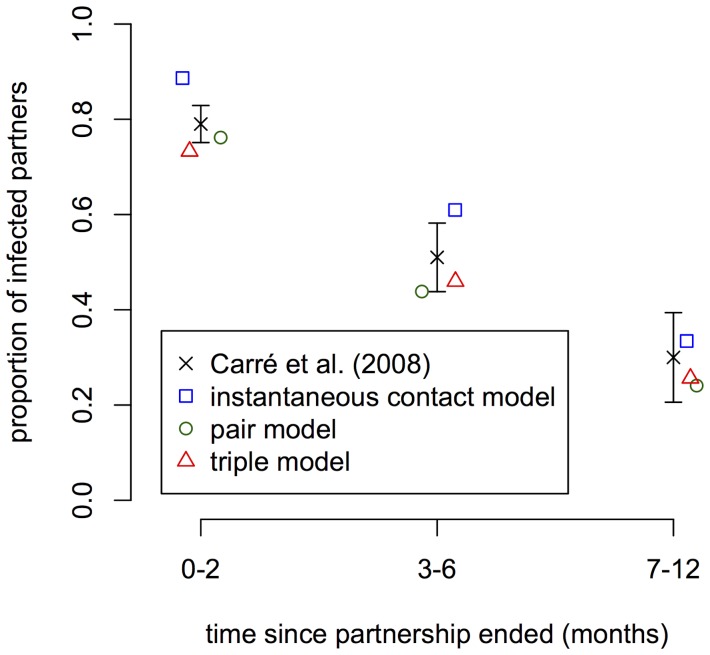
Simulated and empirical data of *C. trachomatis*-positivity in partners of index cases. Black crosses correspond to published data of the proportion of positive partners out of those with a positive test results together with the 95% CI [14]. The other symbols represent simulated data for each of the three different models. In the simulations, it is assumed that the steady-state prevalence of *C. trachomatis* is 3%. Means of 100 simulation runs are shown. Standard errors are small and omitted for better visibility.

At the level of individuals, PN for *C. trachomatis* identifies new index cases. It is therefore important to know how many partners of an index case are infected with *C. trachomatis*. A Swedish study found that the proportion of *C. trachomatis*-positive partners of an index case decreases with increasing duration since they last had sexual intercourse [14]. In the simulations, everyone who is *C. trachomatis*-positive at cross-section is defined as an index case. This corresponds to infected individuals that would be detected through random screening. We can now go through all current and previous partners of an index case and ‘test’ whether or not they are infected. Using the same time periods as in the study by Carré et al. [14], all three models exhibit a similar *C. trachomatis*-positivity of partners with the pair model being within the 95% CI of the data (Fig. 3).

**Figure 4:**
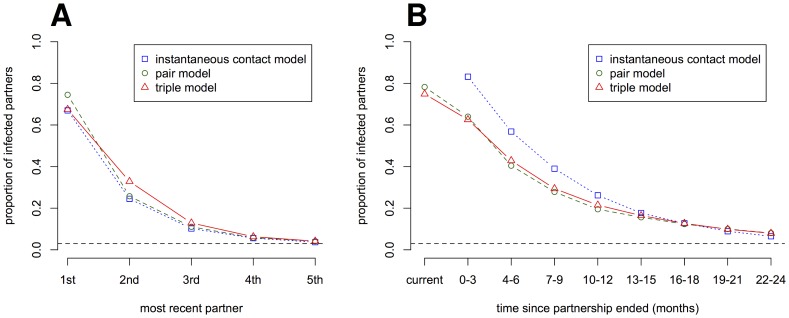
Proportion of *C. trachomatis*-positive partners of index cases. The proportion of partners of an index case who are infected with *C. trachomatis* is shown at a steady-state prevalence of 3% (dashed line). (A) The proportion of infected partners in order of their recency. (B) The proportion of infected partners in order of their breakup date. For each strategy, means of 100 simulation runs are shown. Standard errors are small and omitted for better visibility.

This behavior can be examined in more detail with Rstisim. First, we investigate in order of recency the proportion of partners who are infected (Fig. 4A). The models give consistent results with 67–75% of the most recent partners of an index case being infected with *C. trachomatis*. The less recent a partnership or contact, the lower the probability that the partner is infected. The proportion of infected individuals up to the third most recent partner is still substantially higher than the population prevalence.

We can also group the partners of an index case by the time period since the partnership has ended in greater detail than in the empirical study by Carré et al. [14] (Fig. 4B). The results of the pair and triple models differ from those of the instantaneous contact model. The instantaneous contact model does not have current partners by definition and the pair and triple model result in a lower proportion of infected partners whose partnership has ended within the last year. First, this can be explained through the high per contact transmission probability in the instantaneous contact model. Second, transmission in the pair and triple model can occur before the partnership ends. This makes it possible for the partners to have cleared the infection by the time they will be notified. In all three models, as far back as 18 months, a substantial proportion of partners (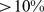) are infected with *C. trachomatis*. This shows that extending PN periods beyond one year yields more new index cases for individual case management than would be found through random screening.

#### Population level effect of partner notification

At the population level, PN can prevent onward transmission of *C. trachomatis* and reduce the overall prevalence of the infection. Here, we investigate the effects of the different PN strategies if they are implemented as part of a population-wide screening program. After the simulations approach the steady-state prevalence of 3%, we introduce random screening of the whole population of young adults. Every woman and man receives screening at a rate of 0.1 per year, i.e., every 10 years on average. If PN is performed, each notified partner will be tested and successfully treated with a probability of 50% [31], [32].

**Figure 5:**
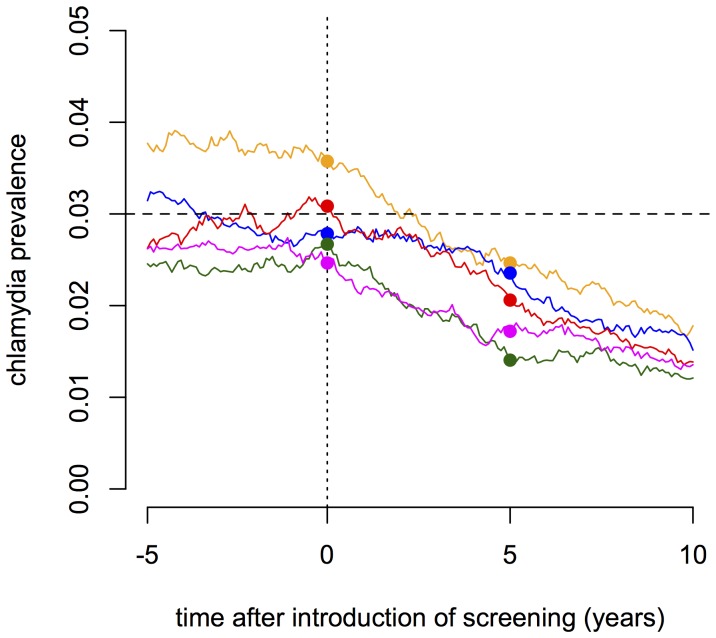
Representative time plots of the prevalence of *C. trachomatis* after the start of a screening intervention. The dots indicate the prevalence at the beginning (dotted line) and after 5 years of the screening intervention. The dashed line indicates the steady-state prevalence of 3% in absence of screening. Every individual receives screening at a rate of 0.1 per year and no partner notification is performed. The colored lines represent five individual simulation runs.

**Figure 6:**
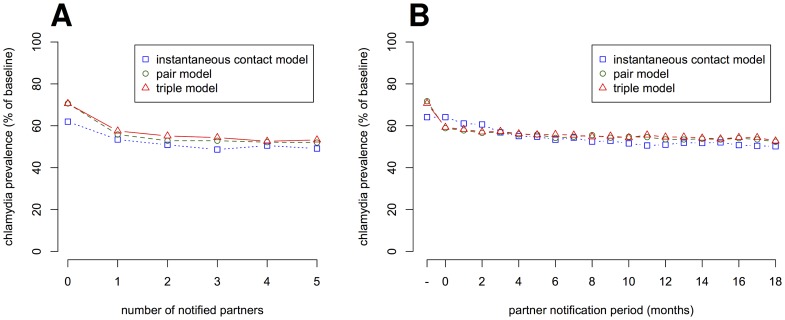
Population level effect of partner notification. The reduction in the prevalence of *C. trachomatis* is given after screening the population for 5 years at a rate of 0.1 per year. (A) The prevalence of *C. trachomatis* for increasing numbers of notified partners, in order of their recency. (B) The prevalence of *C. trachomatis* for different partner notification periods. There is a 50% probability that each notified partner will be tested and successfully treated. For each strategy, means of 100 simulation runs are shown. Standard errors are small and omitted for better visibility.

The simulations show that screening reduces the prevalence of *C. trachomatis* (Fig. 5). After 5 years of screening, assuming that there is no PN, the prevalence is reduced to about 70% of the baseline in the pair and triple model and 60% in the instantaneous contact model (Fig. 6). The effect of screening is smaller in the pair and triple model because index cases in ongoing partnerships engage in sex acts with the same untreated partner after treatment and can be re-infected [6]. This cannot occur in the instantaneous contact model. If PN is performed for at least the most recent partner, the prevalence is reduced to below 60% of the baseline prevalence (Fig. 6A). Increasing the PN period in the instantaneous contact model results in a slight but steady decrease in prevalence (Fig. 6B). However, in the pair and triple model the strongest effect of PN stems from notifying the current partner only. Thus, under more realistic assumptions of sexual partnership dynamics, notification of current partners is sufficient to achieve most of the additional reduction in prevalence at the population level.

### Sensitivity Analyses

#### Heterogeneity in sexual behavior

We have presented results from three models that assume a homogeneous and closed heterosexual population of young adults. The assumption of homogenous mixing provides a good description of the *C. trachomatis*-positivity in partners of index cases (Fig. 3, and refs. [33], [34]). Nevertheless, *C. trachomatis* transmission can be influenced by heterogeneity in sexual behavior, even within a narrow age group. We therefore developed a model where we stratify the population of 16–25 year old women and men into two risk classes (see Fig. S2 in *Text S1* and S3 in *Text S1*). The average number of new heterosexual partners per year is the same as in the models that assume homogeneous mixing. The results from the model with risk classes are consistent with the conclusions drawn from the simpler models. The *C. trachomatis*-positivity in partners from more than 18 months ago is low (Fig. S4B in *Text S1*). Together, this underlines that notifying three or more partners from the last 18 months can be helpful in finding new index cases. Compared to the models that assume homogeneous mixing, the proportion of previous partners of index cases that are infected with *C. trachomatis* is lower for the most recent partner but higher for the third and subsequent partners (Fig. S4A in *Text S1*). The population level effect of PN primarily stems from notifying the most recent partner as for the homogeneous mixing models (Fig. S5A in *Text S1*).

**Figure 7:**
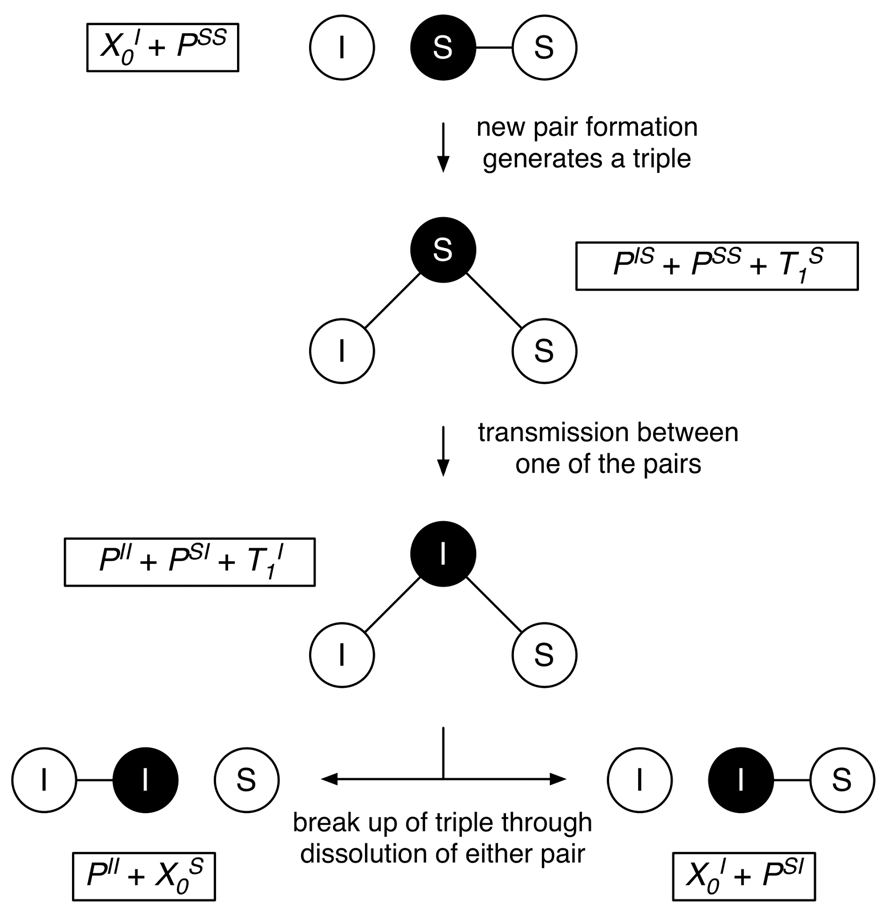
Schematic depiction of the formation and dissolution of a triple. Top: An infected single 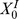 (unconnected white circle) forms a new pair with a susceptible of the opposite sex (black circle in existing pair) which results in a triple 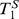 indicating that a susceptible of sex 1 is involved in two pairs. Mid: Transmission can now occur through sexual contacts between the newly formed pair, rendering the triple into 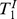. Bottom: The triple 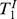 can break up through dissolution of one (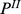) or the other pair (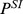). Note that pair formation between one single 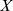 and one pair 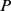 results in two pairs 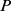 and one triple 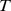 (and vice versa for the pair dissolution). The mathematical representation of each structure is given in rectangles. Subscript 0 denotes white circles (e.g. females) and subscript 1 denotes black circles (e.g. males).

#### Sex-specific differences in duration of infection

The baseline models assume the same disease parameters for women and men. The infectious duration in men is not well established but it has been suggested that it is shorter than in women [35]. We therefore investigate the scenario where the infectious duration in men is half that of in women. We used the pair model for the sensitivity analysis as it incorporates sexual partnerships explicitly while being more tractable than the triple model. Simulations show that our overall conclusions on the effect of PN remain the same (Fig. S6 and S7 in *Text S1*).

#### Differences in uptake of screening and partner notification

The uptake of screening and successful PN are influenced by public health interventions in different settings. The baseline scenario with a screening rate of 0.1 per year is conservative [36], but our simulations show that this can result in a substantial decrease in prevalence if men are screened as well. For the sensitivity analysis, we used again the pair model to investigate the effect of higher screening rates, screening targeted to women only and different probabilities of successful PN (Fig. S8 and S9 in *Text S1*). Higher screening rates and higher probabilities of successful PN result in a more substantial decrease in the prevalence of *C. trachomatis*. The general conclusions about the effects of PN are not altered.

## Discussion

We studied the effects of PN for *C. trachomatis*, both at the level of individuals and the population, using a novel, individual-based modeling framework. The model simulations suggest that, while extending PN periods beyond one year helps to find new index cases, most of the additional effect that PN has on reducing transmission in a general heterosexual population of young adults is achieved by notifying the current or most recent partner.

To our knowledge, this is the first *C. trachomatis* modeling study that investigates the effects of PN of previous partners for realistic look-back periods commonly applied by health care providers. To this end, we developed the modeling framework Rstisim with which one can reconstruct the entire sexual partnership network over any given time period. There are other publicly available software packages that offer some of the features of Rstisim. For example, STI transmission can be simulated using the R package *statnet*, which generates sexual contact networks based on exponential random graph models [37]. Rstisim allows implementation in an individual-based manner of both simple models formulated by ODEs and more sophisticated models where, for example, the behavior of an individual depends on the previous sexual history [38]. Due to this flexibility, we were able to alter and test different assumptions about sexual partnership dynamics. We used three different models in this study, which shows that taking partnerships explicitly into account is a necessary complexity to study the effects of the two PN strategies. The distinction between current and previous partners cannot be made in a model where contacts are assumed to happen instantaneously, but was necessary for understanding the effect of PN on limiting onward transmission in the population. The additional assumption of concurrency (triple model) did not affect our results in this model of a general heterosexual population of young adults.

Our goal was to investigate some general properties of PN for *C. trachomatis* in three basic models that have different assumptions about the sexual partnership dynamics. The models were kept deliberately simple so they can be directly compared and adjusted to exhibit the same numbers of partners and sex acts, capturing the partner change rates that are observed in population-based studies. We thus made some simplifying assumptions. First, we treat the population of 16–25 year olds as a closed population that mixes homogeneously. This assumption probably resulted in realistic dynamics in our study because it was restricted to the age group that drives chlamydia transmission in the wider population. Nevertheless, heterogeneity in sexual behavior can influence the transmission of STIs but the overall findings from a model with two levels of sexual activity in the sensitivity analysis (*Text S1*) were consistent with the simpler models. Second, we did not account for differences in sexual behavior between women and men. We acknowledge that the distribution of women and men between risk groups could differ and men report concurrent partnerships more often than women. Third, we assumed the frequency of sex acts per partnership to be independent of the number of partners in the model with concurrency. We expect that a lower frequency of sex acts per partner for individuals with two partnerships would have a minor effect on the results since the assumption of concurrency did not strongly affect our results in the first place. Fourth, there could also be sex-specific differences in the infection parameters. Our sensitivity analysis showed similar results for the pair model when the infectious duration in men was assumed to be half that in women. The average infectious duration of *C. trachomatis* in men is not well-established, however, and more reliable estimates are needed. Lastly, we did not consider the possibility of temporal immunity against *C. trachomatis* infection after natural clearance. Modeling studies have shown that long lasting immunity can reduce the effect of treatment through screening or PN [6], [39] and even result in a rebound in prevalence [40]. It remains to be determined, however, whether the duration of immunity against *C. trachomatis* infection is long enough to cause these effects [41].

Mathematical and computational models of STI transmission have become increasingly complex over the past two decades. It is possible to incorporate detailed descriptions of sexual behavior and infection characteristics in such models, but this can result in widely different predictions of model outcomes [8], [42]. Such differences can arise because, as model complexity increases, it becomes more difficult to obtain the reliable data for model parameterization. The choice of model complexity should be in balance between incorporating necessary features while keeping the model tractable [43], [44]. Simpler models remain very powerful for studying the impact of public health interventions against STIs [45]. For *C. trachomatis*, simple models can give a good description of the transmission dynamics [39] and provide conclusions in line with more detailed models that include age and risk stratification [8], [46]. In this study, we focused on a quantitative description of the sexual partnership dynamics between young adults, and the pair and triple model appear to describe the observed durations of partnerships and the gaps between them remarkably well.

Our simulation study illustrates that the choice of PN strategy for *C. trachomatis* in a general heterosexual population of young adults depends on the public health context in which it is applied. At the individual level, our results suggest that tracing as many as three partners from the preceding 18 months can be helpful in finding new index cases. This is in line with findings from Sweden [14]. Second, at the population level, PN of current partners of an index case should be prioritized: notifying previous partners has little effect on limiting onward transmission because previous partners of an index case are, if *C. trachomatis*-positive, likely to have been infected for a long time. Hence, there is a high probability that they have already transmitted the infection to other people before they cleared the infection spontaneously. In a scenario where screening is targeted towards high-risk individuals, it is likely that notifying previous partners of index cases would have a stronger effect on limiting onward transmission due to higher partner change rates among these individuals [47].

There are only a few modeling studies that have specifically investigated the effects of PN for *C. trachomatis*. Our findings confirm those of Kretzschmar et al. [9], [10], who also consider a dynamic sexual partnership network but restrict PN to current partners only. PN can render a screening program more effective, especially if screening rates are high (see *Text S1*). Armbruster & Brandeau [18] found that increasing contact tracing capacity results in a substantial reduction of *C. trachomatis* prevalence, although with diminishing returns. They investigate the effects of PN for index cases who seek treatment for symptoms and assume a static sexual network with a relatively high prevalence of *C. trachomatis*. In our study with a dynamic sexual network, previous partners of an index case do not contribute to re-infection which might explain why our study found that notifying previous partners had little additional effect on reducing *C. trachomatis* transmission.

There are several open questions that might be addressed in future studies. As noted above, one could consider targeted screening scenarios towards groups with frequent partner change rates, which might result in different PN recommendations for different groups. It would also be interesting to study the effects of different PN strategies for other bacterial STIs, such as *N. gonorrhoeae* and syphilis. The Rstisim framework will allow researchers to investigate these and other questions in sexual partnership networks with different levels of complexity. This will ultimately lead to a better understanding of the full potential of PN and the identification of the optimal strategies to deal with *C. trachomatis* and other STIs. In this study, we have shown that PN for *C. trachomatis* can have different effects at the level of individuals and at the population.

## Methods

### Individual-based Modeling Framework

Rstisim is a stochastic, individual-based (or agent-based) modeling framework that can simulate the transmission of an arbitrary STI in a sexual partnership network of any level of complexity (see *Text S2* for a brief description). It is written in C++ and can be downloaded at http://www.stat.nus.edu.sg/~staar/rstisim as a package for the R software environment for statistical computing [48]. A key concept of the partnership formation in Rstisim is that every individual can be assigned a contact or pair formation rate at which they will initiate a partnership. Every individual can also accept a partnership that was initiated by another individual. This offers various ways to implement partnership formation rules. For example, it provides the flexibility to implement classical models of STI transmission dynamics that are formulated by ODEs [24], as well as more sophisticated models where contact rates depend on the individual’s previous sexual partnership history. At any point in time, all information on individuals, partnerships and infections can be accessed and the sexual contact network can be graphically depicted using the *network* package [49].

### Modeling *C. trachomatis* Transmission

To investigate the transmission of *C. trachomatis* in a heterosexual partnership network we implemented three different models in Rstisim: an ‘instantaneous contact model’, a ‘pair model’ and a ‘triple model’ where individuals can have concurrent partnerships. We keep the models deliberately simple (assuming homogeneous mixing), which facilitates the derivation of exact parameter values and allows for a direct comparison of the different assumptions about sexual partnership dynamics. Note that Rstisim also allows sexual contacts between men, or between women. We do not consider them for simplicity. Here, we provide the ODEs of the contact and partnership dynamics (and if applicable the transmission dynamics) for all three models. The structure of these models is then directly implemented into Rstisim where event times (partnership formation and dissolution, sex acts and clearance of *C. trachomatis*) are drawn from exponential distributions around mean values (Table 1). We assume a total population size 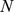 that is equally divided into females and males. The difference in sex is indicated by the subscripts 0 and 1.

#### Instantaneous contact model

Since contacts are assumed to happen instantaneously, individuals always remain single and are denoted by 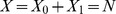. Assuming an SIS-type (susceptible-infected-susceptible) infection [24], the transmission dynamics can be written as follows:
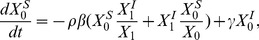
(1)

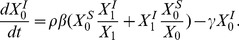
(2)


The superscripts 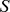 and 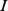 indicate whether individuals are susceptible or infected. The rate at which people have contacts is denoted by 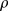 and the transmission probability per contact is given by 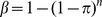 where 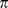 is the transmission probability per sex act and 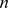 is the number of sex acts per contact. The average duration of an infection is given by 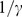. Note that a contact occurs through initiation by single 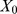 but also if 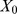 accepts a contact of 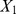.

#### Pair model

We assume that singles 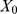 can form a pair, 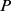, with a single of the opposite sex 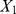 [25]. The transmission dynamics of an STI can then be described as follows:
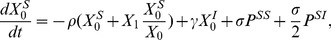
(3)

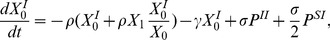
(4)

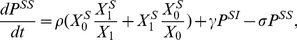
(5)

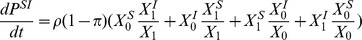


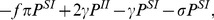
(6)

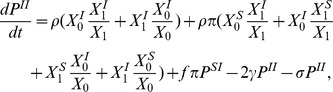
(7)where 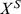 and 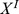 again denote susceptible and infected singles, respectively. Every single 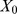 initiates a partnership at a pair formation rate 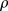 but can also accept a partnership that is initiated by a single 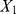. This results in susceptible-susceptible pairs 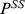, susceptible-infected pairs 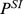 and infected-infected pairs 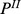. The average duration of a partnership is given by 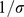. Every partnership begins with an initial sex act where *C. trachomatis* can be transmitted at rate 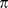. In an ongoing partnership, transmission occurs at rate 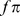 where 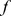 is the frequency of sex acts and 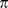 is the transmission probability per sex act.

#### Triple model

The pair model framework can be extended so that individuals can have two sexual partnerships at the same time (concurrency) [50]. This allows us to derive an exact formulation of sexual partnership dynamics, compared to purely stochastic descriptions [51], [52] or moment closure approximations [53], [54]. To account for concurrency, we assume that not only singles 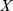 but also individuals that are in one and only one pair 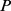 can accept another partnership with a probability 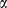 (which is relative to the probability that a single 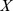 accepts). Such an event can result in a triple 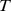 (Fig. 7), which represents the basic unit of concurrent partnerships (an individual with two sexual partnerships at cross-section). Triples can then be elongated to form chains of contacts. Transmission between an infected and susceptible individual happens in the same way as in the pair model. The frequency of sex acts per partnership is constant, i.e., individuals that have two concurrent partnerships have twice as many sex acts per unit of time compared to individuals who are in only one partnership. A full description of the transmission dynamics would require keeping track of the various types of chains together with the infection status of the respective individuals. Here, we concentrate on the overall sexual partnership dynamics that can be described by following the number of singles 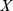, pairs 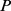 and triples 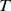 only. The chains are an emergent property of the partnership dynamics and it is therefore not necessary to explicitly consider them in the equations.
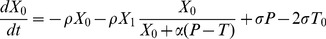
(8)

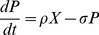
(9)

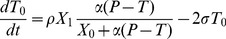
(10)


The total population size is now given by 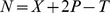 where 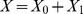 and 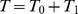. Note that in the term 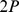, the individuals who form a triple 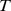 are counted twice and need to be subtracted. We define the level of concurrency at cross-section, 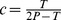, as the ratio of individuals that have more than one partnership to all individuals in a partnership.

#### Sexual behavior data and parameter derivation

To parameterize the heterosexual partnership dynamics, we use data from Britain’s second National Survey of Sexual Attitudes and Lifestyles (Natsal-2), a population-based probability sample survey undertaken between 1999–2001 [26]. In order to directly compare the different models, we adjust the contact or pair formation rate, 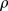, so that each model exhibits the same number of realized contacts or partnerships as given for the group of 16–25 year old women and men in Natsal-2 (Table 1). The contact rate in the instantaneous contact model is half the mean number of new heterosexual partners per year because every individual can be an initiator and an acceptor of a partnership. Assuming the sexual partnership dynamics has approached steady-state, the pair formation rate for the pair model is given by 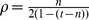, where 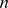 is the average number of new heterosexual partners per year and 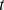 is the total number of heterosexual partners per year. For the triple model, the pair formation rate is given by 
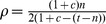
, where 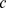 is the level of concurrency. Similarly, the partnership dissolution rate for both the pair and triple model is given by 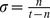. Note that 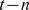 corresponds to the proportion of individuals in a partnership. We also assume the average number of sex acts per partnership to be equal in all three models. The average infectious duration is assumed to be 1 year. This takes into account that most infections are asymptomatic and can persist for more than a year [39], [55]while some infections are shorter due to symptoms. We assume equal transmissibility for both sexes. *C. trachomatis* prevalence rates in 18–24 year olds in Natsal-2 were 3.0% in women and 2.7% in men in Britain as a whole [56]. Similar levels have been observed in young adults in the US, with no significant difference between women and men [57]. In addition, levels of concordance of *C. trachomatis*-positivity in women and men in heterosexual partnerships are very similar [58]. The transmission probability per sex act for each model was calibrated so that the steady state prevalence of *C. trachomatis* was 3%. In the sensitivity analysis, we consider a model where the infectious duration in men is half of that in women, which resulted in a somewhat lower prevalence in men compared to women (see *Text S1*). Data from Natsal-2 were weighted to adjust for unequal selection probabilities and to correct for the age and gender profile in the population, and mean values were taken for women and men together. Parameter solutions were obtained in Mathematica [59]. For the triple model, the desired transmission probability to obtain the steady-state prevalence could only be approximated through numerical simulations. For the definition of gaps and overlaps between partnerships (Fig. S1), we refer to Althaus et al. [8]. Simulation time strongly depends on the population size (see Fig. S10 in *Text S2*) which was set to 20′000 if not otherwise indicated.

## Supporting Information

Figure S1 Gaps and overlaps between sexual partnerships. The emergent gaps and overlaps from the triple model correspond well with population-based data of 16–25 year olds from Natsal-2.
(PDF)

Text S1 Sensitivity analysis. First, this file contains the description of a *Chlamydia trachomatis* transmission model with heterogeneity in sexual behavior (risk class model) together with the effects of screening and partner notification (PN) in this model. Second, the results of screening and PN are shown for the pair model, assuming that the infectious duration in men is shorter than in women. Third, the effects of different rates of screening uptake and probability of PN in the pair model are shown.
(PDF)

Text S2 Description of Rstisim. This file provides a brief description of the individual-based modeling framework Rstisim. It contains examples on how to define the partnership formation and an infection. It also contains a section on simulation times for different models.
(PDF)
