# Supplementary figures and images for: Individual and Population Level Effects of Partner Notification for Chlamydia trachomatis
Source: PLoS One. 2012 Dec 12;7(12):e51438. doi: 10.1371/journal.pone.0051438 (PMC3520891; doi:10.1371/journal.pone.0051438)

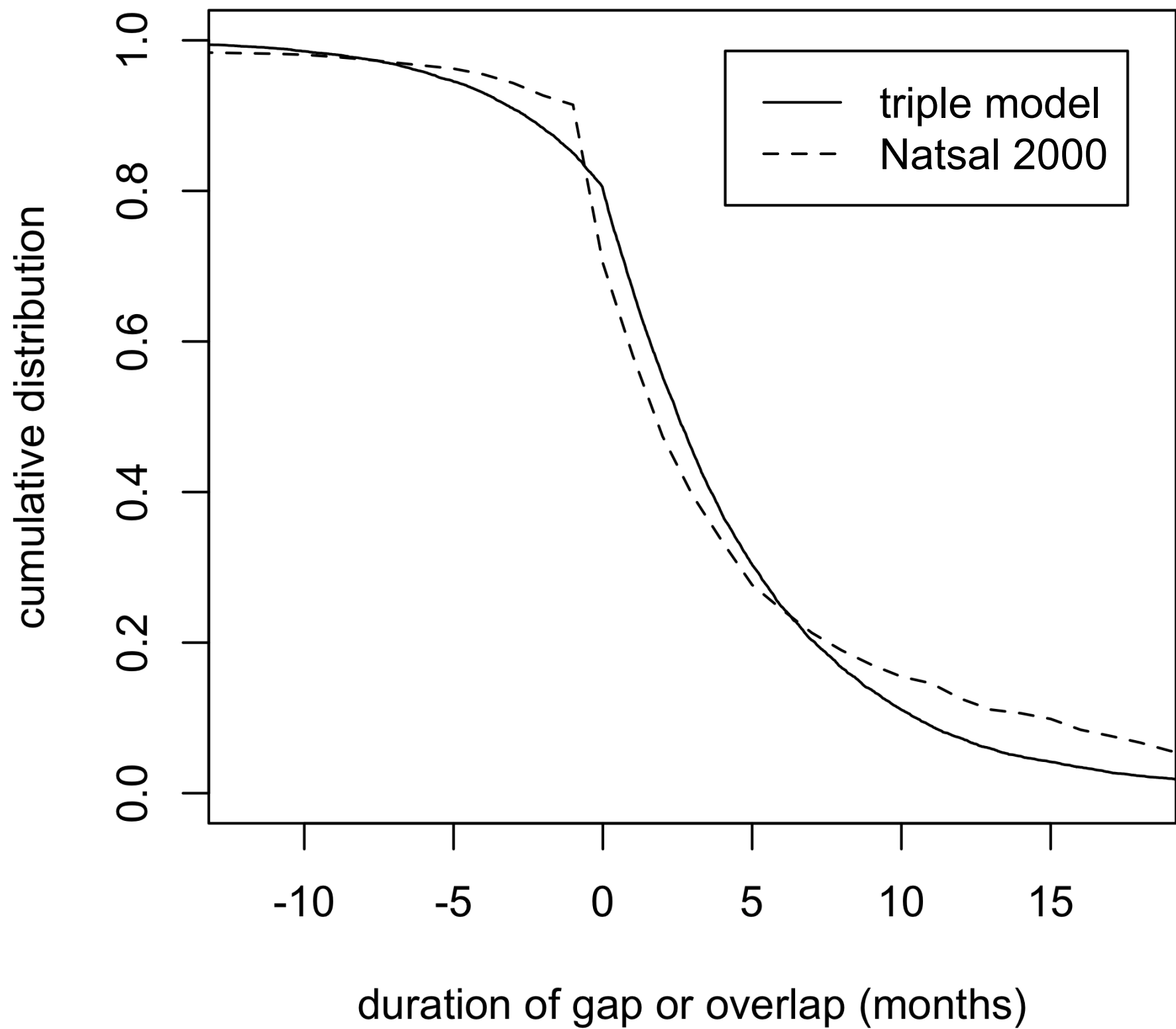

Supplement: Figure S1 — Gaps and overlaps between sexual partnerships. The emergent gaps and overlaps from the triple model correspond well with population-based data of 16–25 year olds from Natsal-2. (PDF) [file pone.0051438.s001.pdf]
