# Supplementary material for: Individual and Population Level Effects of Partner Notification for Chlamydia trachomatis
Source: PLoS One. 2012 Dec 12;7(12):e51438. doi: 10.1371/journal.pone.0051438 (PMC3520891; doi:10.1371/journal.pone.0051438)
Supplement: Text S1 — Sensitivity analysis. First, this file contains the description of a Chlamydia trachomatis transmission model with heterogeneity in sexual behavior (risk class model) together with the effects of screening and partner notification (PN) in this model. Second, the results of screening and PN are shown for the pair model, assuming that the infectious duration in men is shorter than in women. Third, the effects of different rates of screening uptake and probability of PN in the pair model are shown. (PDF) [file pone.0051438.s002.pdf]

Individual and population level effects of partner notification for *Chlamydia trachomatis*

Supporting Information - Text S1: Sensitivity analysis

Christian L. Althaus, Janneke C.M. Heijne,
Sereina A. Herzog, Adrian Roellin, Nicola Low

Overview

In Section 1, we describe a *Chlamydia trachomatis* transmission model with heterogeneity in sexual behavior (risk class model) and show the effects of screening and partner notification (PN). Section 2 shows the results of screening and PN for the pair model (from the main text), assuming that the infectious duration in men is shorter than in women. Finally, Section 3 provides a sensitivity analysis, investigating the effects of different rates of screening uptake and probability of PN in the pair model.

Contents

1 Heterogeneity in sexual behavior **1**
1.1 Risk class model 1
1.2 Individual level effect of partner notification 2
1.3 Population level effect of partner notification 4

2 Sex-specific differences in duration of infection **4**

3 Differences in uptake of screening and partner notification **5**

1 Heterogeneity in sexual behavior

1.1 Risk class model

We develop a model with different sexual activity classes (risk class model) to take into account the heterogeneity in sexual behavior. The notation is based on the instantaneous contact model from the main text. Following Hethcote & Yorke [1] and Garnett et al. [2], we derive the following set of ordinary differential equations:

$$\frac{dX_{i,k}^S}{dt} = \mu X_{i,k} - X_{i,k}^S c_k \sum_{l=1}^{n} \beta_{kl} \rho_{i,kl} \frac{X_{1-i,l}^I}{X_{1-i,l}} + \gamma X_{i,k}^I - \mu X_{i,k}^S, \quad (1)$$

$$\frac{dX_{i,k}^I}{dt} = X_{k,i}^S c_k \sum_{l=1}^{n} \beta_{kl} \rho_{i,kl} \frac{X_{1-i,l}^I}{X_{1-i,l}} - \gamma X_{i,k}^I - \mu X_{i,k}^I. \quad (2)$$

Individuals are denoted by $X_{i,k}$ where the subscript i refers to the sex ($1-i$ being the opposite sex for $i \in \{0,1\}$) and the subscript k refers to one of n sexual risk classes. The superscripts S and I indicate whether individuals are susceptible or infected. Susceptible individuals $X^S_{i,k}$ seek sexual partners at rate c_k and become infected with *Chlamydia trachomatis* by their infected partners $X^I_{1-i,l}$ with a per partnership transmission probability β_{kl}. The average infectious duration is given by $1/\gamma$. $\rho_{i,kl}$ denotes the sexual mixing matrix for an individual of sex i that belongs to risk class k and makes contact to individuals in risk class l. It can be defined as

$$\rho_{i,kl} = \varepsilon\delta_{kl} + (1-\varepsilon)\frac{c_l X_{1-i,l}}{\sum_{m=1}^{n} c_m X_{1-i,m}}, \tag{3}$$

with $X_{1-i,l}$ being the proportion of partners that belongs to sexual risk class l and where δ_{kl} denotes the Kronecker delta (it is equal to 1 if $k = l$ and to 0 otherwise). Mixing can be varied between proportionate ($\varepsilon = 0$) and fully assortative ($\varepsilon = 1$). The duration that individuals remain in this population (16–25 year old) is $1/\mu$, i.e., 10 years.

The model is parameterized with sexual behavior data of 16–25 year old women and men from the UK National Survey of Sexual Attitudes and Lifestyles (Natsal) 2000 [3]. We assume two risk classes ($n = 2$, for low and high sexual activity) and use maximum likelihood estimation to obtain the proportion of individuals in each risk class X_k and the risk-class specific sexual partner change rate c_k [4]. Data for women and men are pooled, i.e., we assume the same sexual behavior for both sexes. Assuming that the realized number of heterosexual partners during one year for individuals of risk class k follows a Poisson distribution with mean c_k, we estimate that 94.6% and 5.4% of individuals belong to the low and high risk class, respectively (Fig. S2A). The corresponding heterosexual partner change rates are 0.61 and 8.05 per year.

We assume that the per partnership transmission probability for contacts between individuals of the low risk class and individuals of the high risk class, $\beta_{12} = \beta_{21}$, is 50%. This is in the range that has been estimated in a medium risk setting [5]. The per partnership transmission probabilities for contacts between two individuals of the low risk class or two individuals of the high risk class are obtained by a scaling factor α, i.e., we define $\beta_{11} = \alpha\beta_{12}$ and $\beta_{22} = \beta_{12}/\alpha$. For $\alpha = 1.9$ and an average infectious duration of 1 year [6, 7], we obtain an endemic *C. trachomatis* prevalence of 3% (β_{11} = 95% and β_{22} = 26.3%). The structure and parameters of the deterministic, population-based model are then implemented in Rstisim in an individual-based manner. Assuming proportionate mixing ($\varepsilon = 0$), we obtain a more clustered sexual contact network (Fig. S2B) compared to a homogeneous population (see main text) and a realistic distribution of *C. trachomatis* infections in the population (Fig. S3). Note that increasing ε towards assortative mixing results in most *C. trachomatis* infections being concentrated among high risk individuals which is not consistent with population-based data from Natsal 2000 [8].

1.2 Individual level effect of partner notification

The *C. trachomatis*-positivity of partners of infected index cases in the risk class model together with the results from the instantaneous contact model and the pair model from the main text is shown in Fig. S4. The *C. trachomatis*-positivity in the most recent partner is lower in the risk class model than the models that assume homogeneous mixing (Fig. S4A) because the transmission probability per partnership in the risk class model is lower. Only contacts between individuals of the low risk class result in a high transmission probability (β_{11} = 95%), which is necessary to obtain the high *C. trachomatis*-positivity of partners as found in the study by Carré et al. [9].The third and subsequent partners are also more likely to be chlamydia positive than the overall population. This is because the infections tend to be concentrated among high risk individuals (Fig. S3), who have higher partner change rates and a higher prevalence of *C. trachomatis* than the overall population. When partners

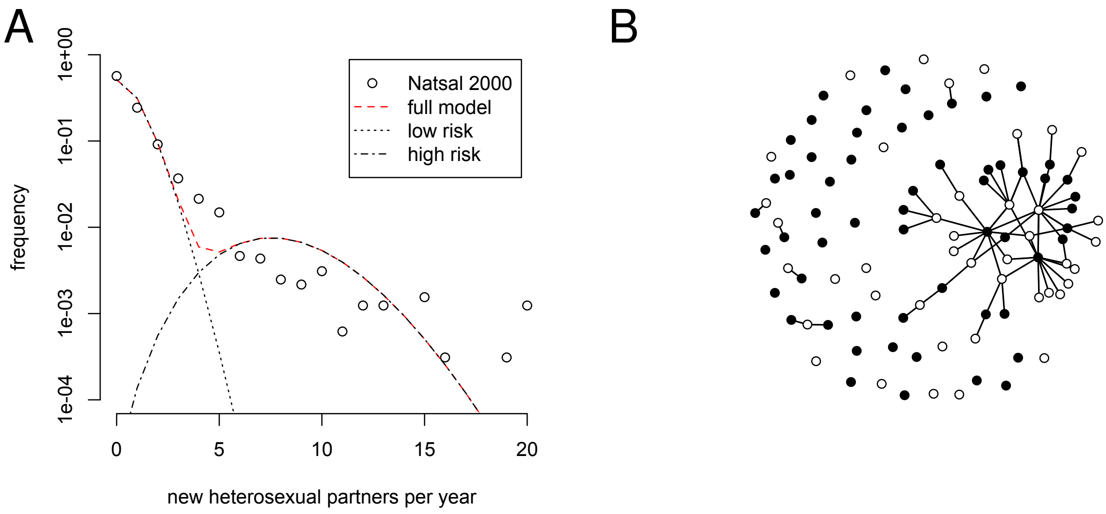

Figure S2: Frequency distribution of the number of heterosexual partners per year and the resulting sexual contact network (A) The numbers of new heterosexual partners per year as reported in Natsal 2000 are shown as circles (pooled data of 16–25 year old women and men). In the risk class model, 94.6% of individuals belong to the low risk class with a partner change rate of 0.61 per year (dotted line) and 5.4% belong to the high risk class with a partner change rate of 8.05 per year (dash-dotted line). The distribution of the number of heterosexual partners per year in the total population of the risk class model is given by the red dashed line. (B) Example of a sexual contact network over a period of 1 year as obtained from the individual-based implementation of the risk class model in Rstisim.

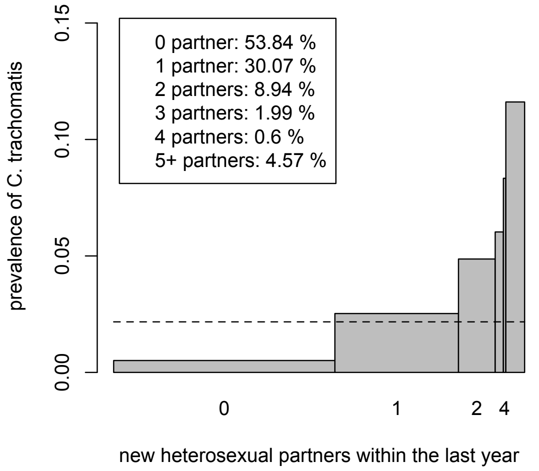

Figure S3: Distribution of *C. trachomatis* infections among individuals with different levels of sexual activity. The width of each bar represents the proportion of people that have had a given number of new heterosexual partners within the last year (the legend shows the distribution of new heterosexual partner numbers). The height of the bar indicates the prevalence of *C. trachomatis* in each group. Note that the overall prevalence (dashed line) can differ from the expected prevalence (3%) due to stochastic fluctuations.

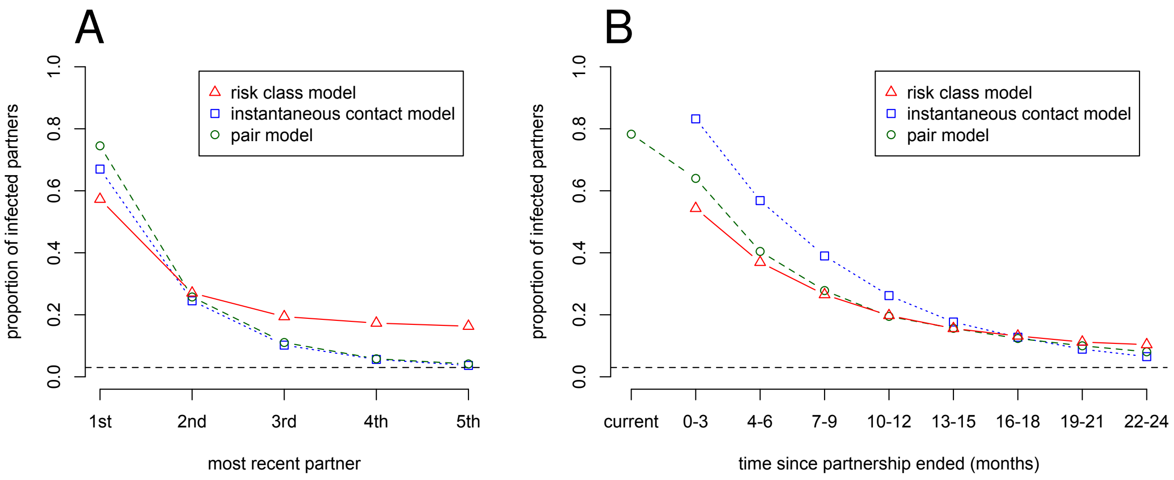

Figure S4: Proportion of *C. trachomatis*-positive partners of index cases in the risk class model together with the results of the instantaneous contact model and the pair model from the main text. The proportion of partners of an index case who are infected with *C. trachomatis* is shown at a steady-state prevalence of 3% (dashed line). (A) The proportion of infected partners in order of their recency. (B) The proportion of infected partners in order of their breakup date. For each strategy, means of 100 simulation runs are shown. Standard errors are small and omitted for better visibility. The population size was set to 20000.

are grouped by the time period since the partnership has ended, *C. trachomatis*-positivity is $>$ 10% as far back as 18 months. The proportion of infected partners in the risk class model is lower than in the instantaneous contact model but similar to the pair and triple models (Fig. S4B).

1.3 Population level effect of partner notification

The effects of screening and PN in the risk class model are shown together with the results from the instantaneous contact model and the pair model from the main text (Fig. S5). As for the pair model, most of the additional reduction in prevalence stems from notifying the most recent partner (Fig. S5A). The effects of different PN periods in the risk class model are very similar to those in the instantaneous contact model that assumes homogeneous mixing. Increasing the PN period results in a slight but steady decrease in prevalence (Fig. S5B). This is because the contact with the most recent partner has usually happened sometime during the past year because neither model explicitly accounts for ongoing (current) sexual partnerships. In summary, our conclusion from the main text, that notifying the most recent partner generates most of the additional effect that PN has on reducing *C. trachomatis* transmission, is robust to the assumption of heterogeneity in sexual activity.

2 Sex-specific differences in duration of infection

This scenario uses the pair model from the main text. We assume that the infectious duration in men (6 months) is half of that in women (12 months) and adjust the transmission probability so that the endemic prevalence of *C. trachomatis* is 3%. A per sex act transmission probability of 25.1% results in a prevalence of 3.1% and 2.9% in women and men, respectively. Our conclusions, both for the *C. trachomatis*-positivity in partners (Fig. S6A) and the population level effect of PN (Fig. S6B) are

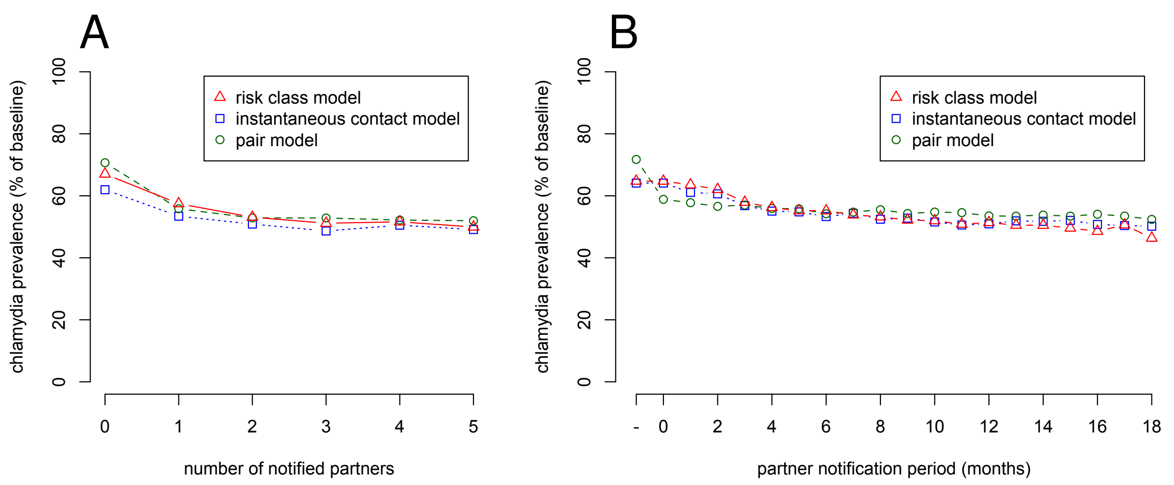

Figure S5: Population level effect of partner notification in the risk class model together with the results of the instantaneous contact model and the pair model from the main text. The reduction in the prevalence of *C. trachomatis* is given after screening the population for 5 years at a rate of 0.1 per year. (A) The prevalence of *C. trachomatis* for different numbers of notified partners. (B) The prevalence of *C. trachomatis* for different partner notification periods. There is a 50% probability that each notified partner will be tested and successfully treated. For each strategy, means of 100 simulation runs are shown. Standard errors are small and omitted for better visibility. The population size was set to 20000.

the same as those drawn from the pair model in the main text.

3 Differences in uptake of screening and partner notification

These scenarios use the pair model from the main text. The prevalence of *C. trachomatis* can be substantially reduced (Fig. S8) at higher screening rates or higher probabilities of successful PN than in the baseline scenario. We also studied the scenario when only women are screened (S9) and find the same behavior. For example, the *C. trachomatis* prevalence can be reduced to about 30% of the pre-screening levels if all women are tested every other year (which corresponds to a screening rate of 0.5 per year) and if 50% of the current partners are notified and successfully treated (S9D).

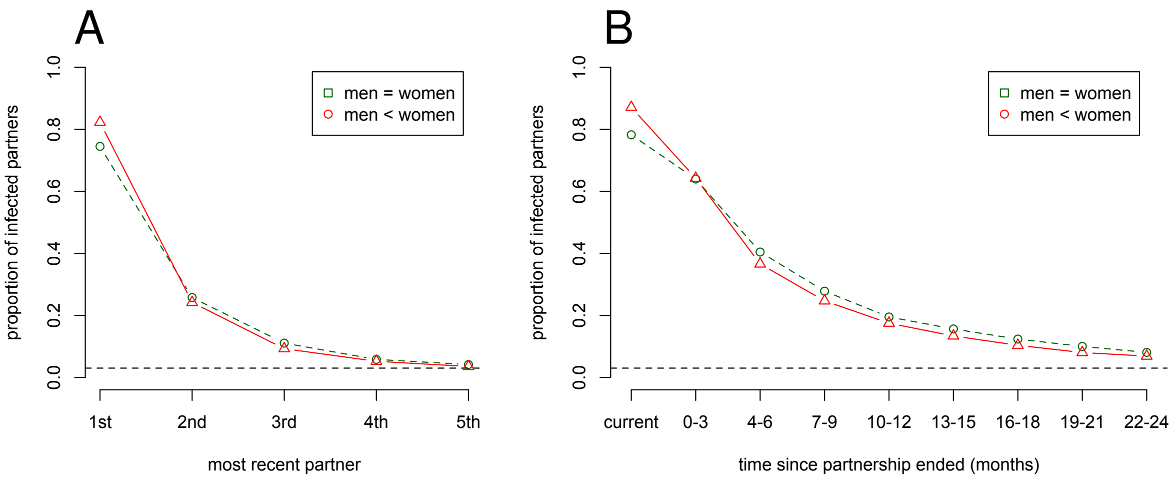

Figure S6: Proportion of *C. trachomatis*-positive partners of index cases in the pair model if the infectious duration in men is shorter than in women. The proportion of partners of an index case who are infected with *C. trachomatis* is shown at a steady-state prevalence of 3% (dashed line). (A) The proportion of infected partners in order of their recency. (B) The proportion of infected partners in order of their breakup date. For each strategy, means of 100 simulation runs are shown. Standard errors are small and omitted for better visibility. The population size was set to 20000.

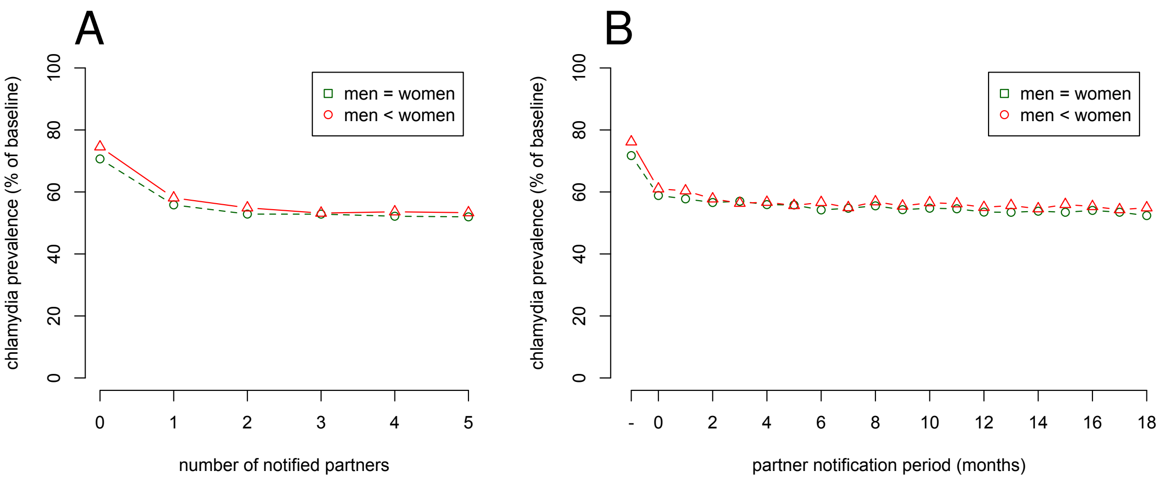

Figure S7: Population level effect of partner notification in the pair model if the infectious duration in men is half of that in women. The reduction in the prevalence of *C. trachomatis* is given after screening the population for 5 years at a rate of 0.1 per year. (A) The prevalence of *C. trachomatis* for different numbers of notified partners. (B) The prevalence of *C. trachomatis* for different partner notification periods. There is a 50% probability that each notified partner will be tested and successfully treated. For each strategy, means of 100 simulation runs are shown. Standard errors are small and omitted for better visibility. The population size was set to 20000.

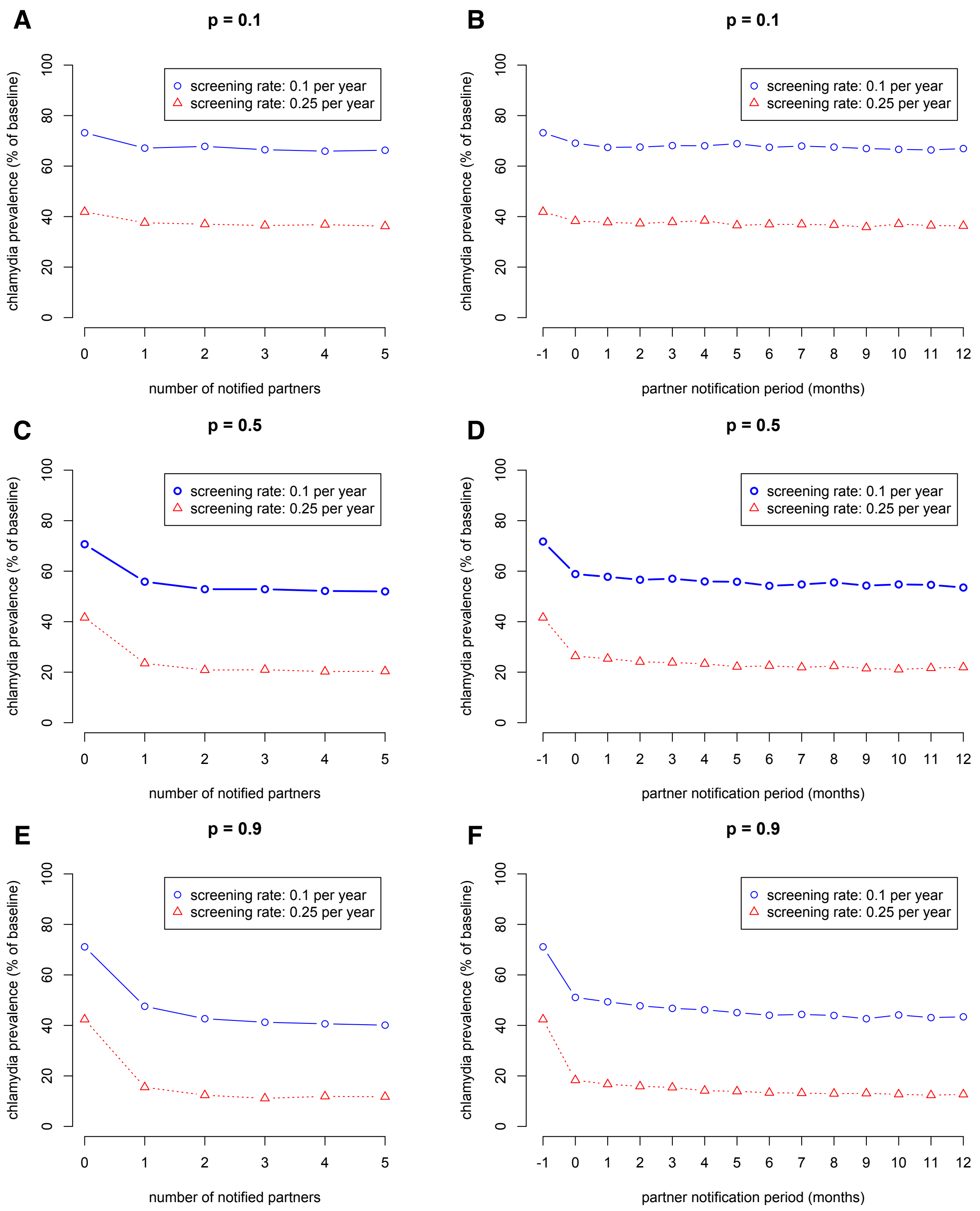

Figure S8: Reduction of *C. trachomatis* prevalence for different screening (women and men) and partner notification scenarios in the pair model. Left panels: Prevalence of *C. trachomatis* for different numbers of notified partners. Right panels: Prevalence of *C. trachomatis* for different partner notification periods. The probability that a notified partner is tested and successfully treated is 10% (A and B), 50% (C and D) and 90% (E and F). All women and men are screened as indicated. All other parameters are as given in the main text. The thick lines correspond to the results from the pair model that are given in the main text.

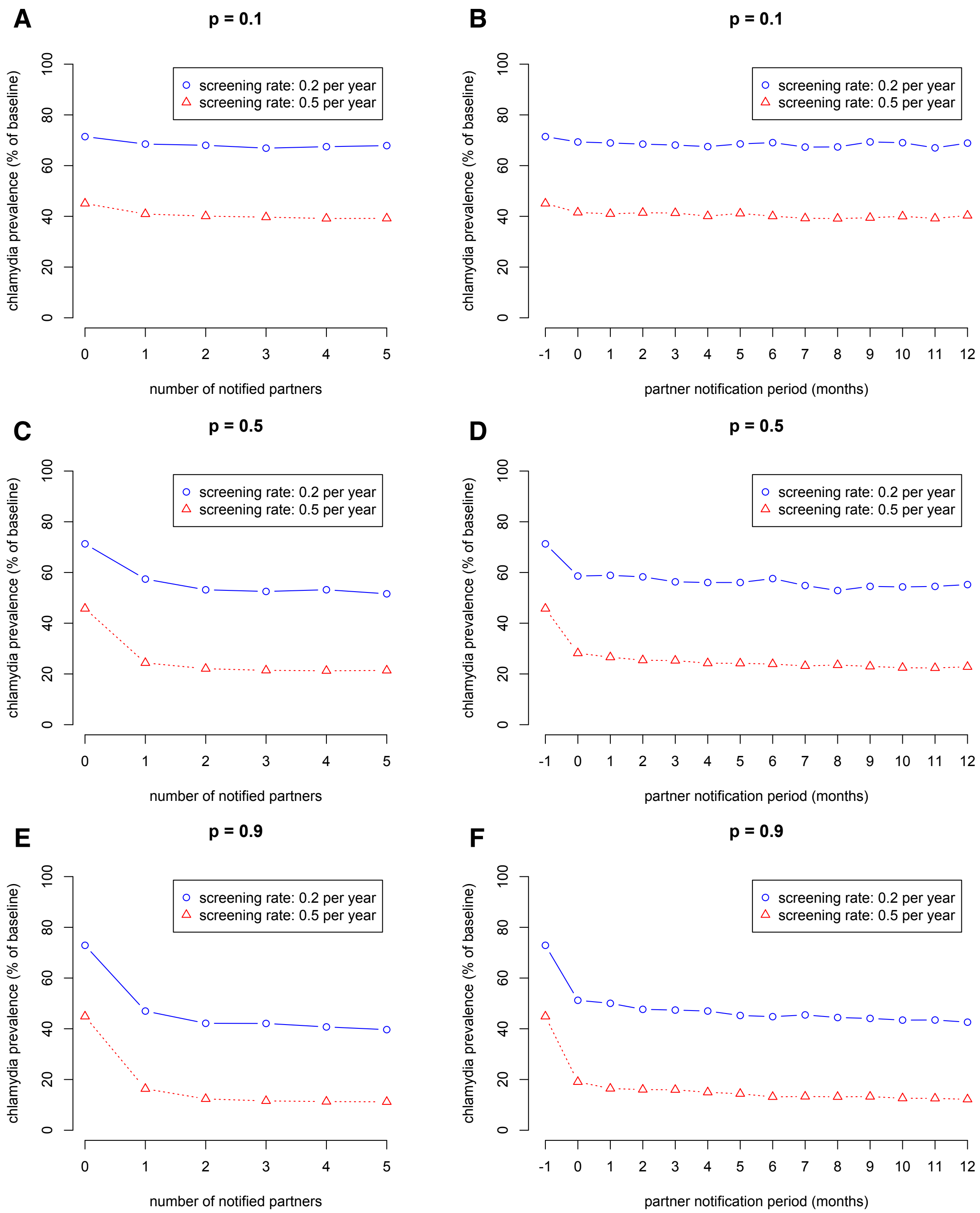

Figure S9: Reduction of *C. trachomatis* prevalence for different screening (only women) and partner notification scenarios in the pair model. Left panels: Prevalence of *C. trachomatis* for different numbers of notified partners. Right panels: Prevalence of *C. trachomatis* for different partner notification periods. The probability that a notified partner is tested and successfully treated is 10% (A and B), 50% (C and D) and 90% (E and F). Only women are screened with rates as indicated. All other parameters are as given in the main text.

References

1. Hethcote HW, Yorke JA (1984) Gonorrhea transmission dynamics and control. Lecture Notes in Biomathematics. Springer-Verlag, Berlin.

2. Garnett GP, Mertz KJ, Finelli L, Levine WC, St Louis ME (1999) The transmission dynamics of gonorrhoea: modelling the reported behaviour of infected patients from Newark, New Jersey. Philos Trans R Soc Lond B Biol Sci 354:787–97. doi:10.1098/rstb.1999.0431.

3. Johnson AM, Mercer CH, Erens B, Copas AJ, McManus S, et al. (2001) Sexual behaviour in Britain: partnerships, practices, and HIV risk behaviours. Lancet 358:1835–1842. doi:10.1016/S0140-6736(01)06883-0.

4. Bolker BM (2008) Ecological Models and Data in R. Princeton, NJ: Princeton University Press. ISBN: 0691125228.

5. Althaus CL, Heijne JCM, Low N (2012) Towards More Robust Estimates of the Transmissibility of Chlamydia trachomatis. Sex Transm Dis 39:402–4. doi:10.1097/OLQ.0b013e318248a550.

6. Althaus CL, Heijne JC, Roellin A, Low N (2010) Transmission dynamics of Chlamydia trachomatis affect the impact of screening programmes. Epidemics 2:123 – 131. doi:10.1016/j.epidem.2010.04.002.

7. Heijne JCM, Althaus CL, Herzog SA, Kretzschmar M, Low N (2011) The role of reinfection and partner notification in the efficacy of Chlamydia screening programs. J Infect Dis 203:372–7. doi:10.1093/infdis/jiq050.

8. Althaus CL, Turner KME, Schmid BV, Heijne JCM, Kretzschmar M, et al. (2012) Transmission of Chlamydia trachomatis through sexual partnerships: a comparison between three individual-based models and empirical data. J R Soc Interface 9:136–146. doi:10.1098/rsif.2011.0131.

9. Carré H, Boman J, Osterlund A, Gärdén B, Nylander E (2008) Improved contact tracing for Chlamydia trachomatis with experienced tracers, tracing for one year back in time and interviewing by phone in remote areas. Sex Transm Infect 84:239–42. doi:10.1136/sti.2007.028068.
