# Supplementary material for: Individual and Population Level Effects of Partner Notification for Chlamydia trachomatis
Source: PLoS One. 2012 Dec 12;7(12):e51438. doi: 10.1371/journal.pone.0051438 (PMC3520891; doi:10.1371/journal.pone.0051438)
Supplement: Text S2 — Description of Rstisim. This file provides a brief description of the individual-based modeling framework Rstisim. It contains examples on how to define the partnership formation and an infection. It also contains a section on simulation times for different models. (PDF) [file pone.0051438.s003.pdf]

Individual and population level effects of partner notification for *Chlamydia trachomatis*

Supporting Information - Text S2: Description of Rstisim

Christian L. Althaus, Janneke C.M. Heijne,
Sereina A. Herzog, Adrian Roellin, Nicola Low

Overview

This file provides a brief description of the individual-based modeling framework Rstisim. It contains examples on how to define the partnership formation and an infection. It also contains a section on simulation times for different models.

Rstisim is an individual-based (or agent-based) model simulation software written in C++ and is fully integrated into the statistical software package R [1]. Its main purpose is to simulate STI-type of infections, which involve formation of complex dynamic partnership networks. Some of the internal structure of Rstisim was inspired by a model developed for the Chlamydia Screening Studies (ClaSS) project [2], which was designed to investigate the effectiveness and cost-effectiveness of systematic screening for *Chlamydia trachomatis* infections. We refer to Garnett et al. [3] for a survey on general modeling techniques, including individual-based models, and to Althaus et al. [4] for STI-specific model comparisons.

The software is event-based and runs in continuous time. At the heart is a so-called *event-queue* that stores events in form of a *binary heap* [5]. This structure allows for fast insertion of new events at any time point and fast access to the root, which represents the event to be executed next. These are the only two operations needed for our purpose (deleting arbitrary events from the queue is done by flagging them as *deleted*). Event-based simulations (EBS) differ from the popular discrete time, day-by-day simulations (DBDS, the time steps can be others than days, of course). In DBDS, the occurrences of events are regulated by per-day probabilities of these events happening (see e.g. Low et al. [2]), which may be intuitive from the modeling point of view, but is rather wasteful in terms of CPU time, as the potential occurrence of each possible event has to be simulated each day for each object. It is clear that, especially for rare events, this is not optimal. Even if events (such as death of an agent, end of partnerships) are simulated far in advance, the software has to query every day whether or not the event is due on that specific day. In EBS, the time points of occurrences of events are simulated in advance and stored in the event queue, and, as events are executed, new events are created and stored in the queue. The drawback of this approach is that it can add considerable complexity to the software because future events need to be re-evaluated due to changes in the states of the agents so these events have to be deleted and re-sampled correctly. The pay-off is increased speed: once a future event has been determined and it has been stored in the event-queue, there is no need to enter this subroutine again, until the event actually occurs. However, the intuitive per-day probabilities have to be replaced by a distributional approach, that is, all events

have a certain distribution of when they occur in the future. In EBS, the analogue of a constant per-day probability is the exponential distribution and events with changing per-day probabilities can be simulated using a combination of time inhomogeneous Poisson processes and thinning [6] and the well-known rejection algorithm [7].

The different (simplified) conceptual layers of Rstisim consist of

- Agents (birth/death);
- Partnerships (formation/dissolution, any number of concurrent partnerships possible);
- Sexual contacts (protected/unprotected, within partnerships only);
- Infections (transmission during sexual contacts);
- Intervention (visit to health care setting due to symptoms or due to screening);
- Partner notification / contact tracing.

Agents, partnerships and infections (let us call them *objects* from now on) can be configured to be of any finite number of *types* (such as gender for agents, casual/steady partnerships); types are fixed during the lifetime of an object. Objects can also be in any finite number of *states* (e.g. an infection may be programmed to be *latent*, *symptomatic* and *asymptomatic*); states can change during the lifetime of an object. The flexibility of Rstisim comes from the fact that the types and states can be freely configured by the user and that the time points of any future events can depend on the types and current states of the agents, partnerships and infections at the time when the future time point is decided (that is, sampled). Furthermore, these time points might also depend on built-in variables (current number of partners, distance from the index case in a sequence of partner notifications, number of treatments for an infection that an agent has undergone, etc.).

We give three short examples of the capabilities of Rstisim below. A full description of the functionality of the software is not possible here. A full model is defined in one configuration file, which is read and interpreted by the R part of the package in order to initialize the C++ code. The simulation can be run repeatedly any fixed length of time. After that, the user can retrieve various data matrices describing the current individual states of the agents, partnerships, infections etc. From these basic data matrices, more complex statistics such as detailed partnership networks and partner notification cascades can be reconstructed. As all the data matrices are stored in native R formats one can make full use of all the built-in functionality and extensions of R, which simplifies and speeds up the analysis.

The partnership formation rule currently implemented is that from the ClaSS model [2]. Assume there are n agents in the population. Once an agent (initiator) decides to start a partnership that agent will form a new partnership at that time point. The software will then find a partner among all the other (n-1) agents proportionate to (n-1) weights that depend on the initiator and each respective individual. In order to avoid calculating all the weights, the rejection algorithm is used again: assume that the weights are standardised to be less or equal to one; pick uniformly an agent among the (n-1) remaining ones, calculate the weight w based on the initiator and the chosen agent, then toss a coin with probability w of showing heads. If the coin shows heads, take that agent as the partner, otherwise repeat the loop. Two examples of how weights are assigned to potential partners are given in *Configuration Example 1* and *2*.

Configuration Example 1: Definition of the partnership formation in a model with concurrency

```
1  partnershipformers: {
2    individual = {
3      active = ["female","male"];
4      seek = { depends="currentpartners";
5        0 : { rate = 1.34544/1y; };
6        1 : never;
7      };
8      accept = { depends="currentpartners";
9        0 : 1;
10       1 : 0.294195;
11       2 : 0;
12     };
13     mixingfactors = {
14       names=["f2m","m2f"];
15       f2m = { from="female"; to="male"; value=1; };
16       m2f = { from="male"; to="female"; value=1; };
17     };
18   };
19 };
```

In Line 3 we declare that both males and females should actively initiate partnerships. The `seek` parameter defines at which time points this should happen (it corresponds to the pair formation rate ρ in the triple model from the main text). In the above example, initiating partnerships will only happen if the individual is single (that is, if the internal variable `currentpartners` is zero). If this is the case, then the next time point of initiating a partnership will be an exponentially distributed time with mean 1.35 years away from the current simulation time (Line 5); the first such event is set when the individual is created (that is, "born"). The `accept` and `mixingfactors` are used to calculate the weights between the initiator and the potential partner. If the potential partner has currently no partners, then the base weight is 1, if it has 1 partner it is 0.29 (corresponding to α in the triple model from the main text) and if it has more partners it will always be 0. This number is then multiplied by `mixingfactors`, which ensures that only heterosexual partnerships are created (that is, if both individuals have the same sex, then the `mixingfactors` parameter will be 0, otherwise it will be 1). Note that if an individual accepts a partnership, then its next partnership initiation event defined via the `seek` parameter will automatically be re-evaluated (in the above case just deleted, as individuals who are in a partnership do not initiate as defined on line 6). If the person becomes single again, the seek parameter is again used to install a new partnership initiation event in the future.

Configuration Example 2: Definition of the partnership formation in a model with risk classes

```
partnershipformers: {
  individual = {
    active = ["female","male"];
    seek = { bybin;
    low : { rate = 0.6078578/1y; };
      high : { rate = 8.0517338/1y; };
  };
  seekfactor = 0.5;
    mixingfactors = {
```

```
10        symmetric = "type";
11        names = ["female2malelow","female2malehigh"];
12          female2malelow = {from="female"; to={type="male";bin="low";};
13             value = {bybin; low=0.07549403; high=0.07549403;};};
14          female2malehigh = {from="female"; to={type="male";bin="high";};
15             value = {bybin; low=1.0; high=1.0;};};
16      };
17    };
18  };
```

In Line 4 we declare the rate at which individuals seek their partners. The heterosexual population is stratified into two risk classes (`low` and `high`) that have their specific contact rates as given in the risk class model (see *Text S1*). The contact rates are scaled with 0.5 (`seekfactor`) because an individual can both be an initiator and acceptor of a partnership (Line 8). Potential partners are randomly chosen from the population and weighted depending on the risk class of the initiator and the acceptor (`mixingfactors`). The mixing is symmetric according to `type`, i.e., it is the same if a female chooses a male and vice versa. The values are interpreted as follows: if a female of risk class `low` seeks a partner and the algorithm samples a male of risk class `low`, the probability that this partnership will be accepted (its weight) is 0.075 (Line 13). If the algorithm samples a male partner of risk class `high` for the same female, the partnership will be immediately accepted because the weight is 1.0 (Line 15). The values in `mixingfactors` are derived from the mixing matrix of the risk class model (see *Text S1*) through division by the proportion of individuals in the risk group of the acceptor and scaling so that the highest value is 1 (the probability that a partnership is accepted if both partners belong to risk class `high`, Line 15).

Configuration Example 3: Definition of *Chlamydia trachomatis* infection

```
 1  infections : {
 2    chlamydia : {
 3      states = {
 4        names = ["latent", "asymptomatic", "cleared", "treated"];
 5        transitions = {
 6          names = ["l2a","a2c"];
 7          a2c : { from="latent"; to="asymptomatic"; at=10d; };
 8          a2c : { from="asymptomatic"; to="cleared"; at={rate=1/1y;};};
 9        };
11      };
12      prevalencewhenpopulating = 0.03;
13      infectiousness = {bystate; latent=0.01; asymptomatic=0.0895; *=0;};
14    };
15  };
```

In line 4, we define four states the infection can be in: `latent`, `asymptomatic`, `cleared` and `treated` (for the simulations in the main text, we did not use `latent` but it is included here for clarity). By default, an infection will always start in the first state, here `latent`. Line 7 defines a transition from `latent` to `asymptomatic` after exactly 10 days since the infection was created. Line 8 defines a transition from `asymptomatic` to `cleared` (which means naturally cleared). The part "`rate=1/1y;;`" means that after an exponentially distributed time with mean 1 year the infection will change into state `cleared`. By default, the infection will be detached from the agent but will remain in the memory for later analysis via R. Note that there is no natural transition from

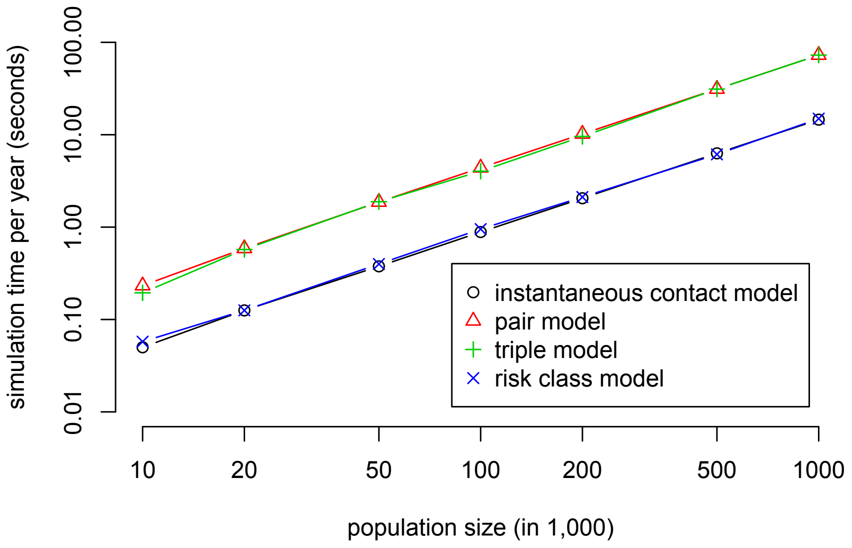

Figure S10: Simulation times for different population sizes. The four different models were simulated on the Amazon Elastic Compute Cloud (Amazon EC2, 64bit Linux, m2.2xlarge, 34.2 GB of memory, one core has 3.25 EC2 Compute Units,which corresponds to about 3.5 GHz Intel Xeon Core). Note the double logarithmic scale.

`asymptomatic` to `treated`. This transition can only be invoked by visiting a health care setting and hence is defined elsewhere in the configuration file. The infectiousness of the infection depends on the state as defined on Line 13: if the infection is in `latent` state, the probability per unprotected sex-act is 0.01. Once the infection proceeds to the `asymptomatic` state, the probability increases to 0.09; otherwise it is 0 (which is not relevant here, as for the two remaining states the infection is removed from the host anyway).

Simulation time

Simulation times in Rstisim heavily depend on the model complexity (Fig. S10). Models where individuals engage in repeated sex acts within partnership (pair and triple model from the main text) require substantially more simulation time than models where sex contacts are assumed to happen instantaneously (instantaneous contact model from the main text and the risk class model from *Text S1*). There is an almost linear relationship between simulation time and population size; 100 simulations runs with a population of 20,000 take about the same time as 10 simulations with a population size of 200,000. However, memory requirements increase with increasing population size. We chose a population size of 20,000 for the simulations in the main text and in *Text S1* because it ensures that the infection does not die out while 100 simulation runs yield acceptably small standard errors of the results. Robust estimation of multiple parameters for complex models is not feasible with current simulation times.

References

1. R Development Core Team (2009) R: A Language and Environment for Statistical Computing. R Foundation for Statistical Computing, Vienna, Austria. ISBN 3-900051-07-0.

2. Low N, McCarthy A, Macleod J, Salisbury C, Campbell R, et al. (2007) Epidemiological, social, diagnostic and economic evaluation of population screening for genital chlamydial infection. Health Technol Assess 11:iii–iv, ix–xii, 1–165.

3. Garnett GP (2002) An introduction to mathematical models in sexually transmitted disease epidemiology. Sex Transm Infect 78:7–12.

4. Althaus CL, Turner KME, Schmid BV, Heijne JCM, Kretzschmar M, et al. (2012) Transmission of Chlamydia trachomatis through sexual partnerships: a comparison between three individual-based models and empirical data. J R Soc Interface 9:136–146. doi:10.1098/rsif.2011.0131.

5. Atkinson MD, Sack JR, Santoro N, Strothotte T (1986) Min-max heaps and generalized priority queues. Commun ACM 29:996–1000. doi:http://doi.acm.org/10.1145/6617.6621.

6. Kallenberg O (1986) Random measures. Contributions to the Study of Art and Architecture. Akademie-Verlag.

7. Press WH, Teukolsky SA, Vetterling WT, Flannery BP (1992) Numerical Recipes in C: The Art of Scientific Computing. New York, NY, USA: Cambridge University Press.
